# Involvement of IL‐17A‐producing TCR γδ T cells in late protective immunity against pulmonary *Mycobacterium tuberculosis* infection

**DOI:** 10.1002/iid3.121

**Published:** 2016-08-24

**Authors:** Masayuki Umemura, Yuko Okamoto‐Yoshida, Ayano Yahagi, Seigo Touyama, Susumu Nakae, Yoichiro Iwakura, Goro Matsuzaki

**Affiliations:** ^1^ Molecular Microbiology GroupDepartment of Infectious DiseasesTropical Biosphere Research CenterUniversity of the Ryukyus1 SenbaruNishiharaOkinawa903‐0213Japan; ^2^ Department of Host DefenseGraduate School of MedicineUniversity of the Ryukyus1 SenbaruNishiharaOkinawa903‐0213Japan; ^3^ Laboratory of Systems BiologyCenter for Experimental Medicine and Systems BiologyInstitute of Medical ScienceUniversity of Tokyo4‐6‐1 ShiroganedaiMinato‐kuTokyo108‐8639Japan; ^4^ Division of Experimental Animal ImmunologyCenter for Animal Disease ModelsResearch Institute for Biomedical SciencesTokyo University of ScienceChiba278‐0022Japan

**Keywords:** IL‐17A, infectious diseases, lung inflammation, γδ T cells

## Abstract

**Introduction:**

Interleukin (IL)‐17A is a cytokine originally reported to induce neutrophil‐mediated inflammation and anti‐microbial activity. The CD4^+^ T cells, which produce IL‐17A, have been well characterized as Th17 cells. On the other hand, IL‐17A‐producing TCR γδ^+^ T cells have been reported to participate in the immune response at an early stage of infection with *Listeria monocytogenes* and *Mycobacterium bovis* in mice. However, the involvement of IL‐17A in protective immunity was not clearly demonstrated in the chronic stage of *M. tuberculosis*‐infected mice.

**Methods:**

We analyzed role of IL‐17A in host defense against chronically infected *M. tuberculosis* using IL‐17A KO mice.

**Results:**

We found that TCR γδ^+^ T cells are a primary source of IL‐17A, but that mycobacterial antigen‐specific Th17 cells were hardly detected even at the chronic stage of *M. tuberculosis* infection. IL‐17A‐deficient mice showed a decreased survival rate, and increased bacterial burden in the lungs after the infection when compared to the wild‐type mice. Furthermore, a histological analysis showed an impaired granuloma formation in the infected lungs of IL‐17A‐deficient mice, which was considered to be due to a decrease of IFN‐γ and TNF at the chronic stage.

**Conclusion:**

Our data suggest that the IL‐17A‐producing TCR γδ^+^ T cells, rather than the Th17 cells, in the infected lungs are an indispensable source of protective immunity against *M. tuberculosis* infection.

## Introduction

Tuberculosis caused by *Mycobacterium tuberculosis* is a typical example of a mycobacterial infection and remains a worldwide threat to health. According to the World Health Organization (WHO) 2015 report, 30% of the world's population is infected with *M. tuberculosis*. This disease generates 9.6 million new patients and 1.5 million deaths every year 1. In addition, the expansion of multidrug‐ and extensive drug‐resistant tuberculosis has thus resulted in a situation that continues to worsen 2. To control drug‐resistant strains of *M. tuberculosis*, it is important to understand the host protective immune response to the infection as this will allow for the development of better vaccines that promote protective responses and limit the pathological effects of immunity.

It is well known that IFN‐γ‐producing Th1 cells protect against exogenous microbes, whereas interleukin (IL)‐4‐ or IL‐13‐producing Th2 cells work against parasites 3. In 2005, the existence of a Th17 subset of cells was established, which are CD4^+^ T cells that secrete IL‐17A 4, 5. IL‐17A initiates inflammation via neutrophils through its interaction with the IL‐17A receptors expressed on various cells, including endothelial cells, epithelial cells, and fibroblasts. The interaction between IL‐17A and IL‐17R induces the expression of IL‐6, G‐CSF, CXCL8/IL‐8, and/or MIP‐2. A recent study showed the synergistic action of IL‐17A with IL‐22 in promoting the production of antimicrobial peptides such as β‐defensin 2, or molecules from the S100 protein family 6. In addition, IL‐17A is not only a mediator of inflammation, but also regulates antigen‐specific adaptive immunity. However, the mechanism underlying the regulation of acquired immunity by IL‐17A‐producing cells is still not clear.

The importance of the IL‐17A produced by TCR γδ^+^ T cells, rather than Th17 cells has been reported in mycobacterial protection 7, 8, 9, 10. We previously reported that the major murine IL‐17A‐producing cells in the *M. bovis* BCG‐infected lungs were TCR γδ^+^ T cells at a relatively early stage of the infection 8, 9, 10. In addition, IL‐17A positively regulated infection‐induced mature granuloma formation in the *M. bovis* bacillus Calmette–Guérin (BCG)‐infected lungs 9, 10. Furthermore, the expression of IL‐17A in the early phase of infection is necessary for the Th1 type delayed‐type hypersensitivity response and optimum granuloma formation. Importance of IL‐17A in Th1‐mediated protective immunity against mycobacterial infection was also indicated in human RORC mutant patients 11. RORC encodes RORγ (t) transcription factor which is indispensable in development of IL‐17A‐producing cells, and hereditary congenital deficiency of the RORγ resulted not only in lack of Th17 and other IL‐17A‐producing cells, but also decrease of mycobacteria‐specific Th1 cells and life‐threatening infection of low‐virulent *Mycobacterium bovis* BCG vaccine strain. Therefore IL‐17A might be required to regulate cell‐mediated adaptive immunity.

In the present study, we analyzed the involvement of IL‐17A in the protective immune response against chronic *M. tuberculosis* infection. Our results demonstrated that TCR γδ^+^ T cells were the major IL‐17A‐producing cells involved during the chronic stage of *M. tuberculosis* infection. IL‐17A‐deficient mice showed decreased survival rates during the 1‐year observation period, and had an increased bacterial burden from day 30 of the infection when compared to wild‐type C57BL/6 mice. Furthermore, the lack of IL‐17A resulted in impaired granuloma formation at the chronic stage of *M. tuberculosis* infection. These data suggest that the IL‐17A produced by TCR γδ^+^ T cells is an important cytokine involved in the protective immunity against chronic *M. tuberculosis* infection even when acquired immunity is established.

## Results

### IL‐17A is required for the host defense against *M. tuberculosis* infection

**Figure 1:**
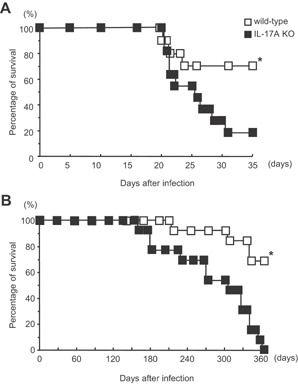
The survival of IL‐17A KO mice after mycobacterial infection. Groups of 11–13 mice were infected i.t. with 1 × 10^5^ (A) or 1 × 10^3^ (B) CFU of *M. tuberculosis* H37Rv, and the survival rates were monitored. The statistical significance of the differences in survival was determined by the generalized Wilcoxon's test. *P *= 0.0018 (A) and *P *= 0.0052 (B), respectively. The asterisk (*) indicates that the difference was considered to be significant.

We first examined the survival rate of IL‐17A KO mice after intratracheal (i.t.) infection with *M. tuberculosis*. Almost all of the IL‐17A KO mice died between days 21 and 35 after high‐dose (1 × 10^5^ cfu) *M. tuberculosis* pulmonary infection, whereas more than 70% of wild‐type C57BL/6 mice survived beyond this stage (Fig. 1A). In the case of infection with a low dose (1 × 10^3^ cfu) of *M. tuberculosis*, all IL‐17A KO mice died by 1 year after the infection, whereas approximately 70% of the infected wild‐type C57BL/6 mice survived (Fig. 1B). As a result, IL‐17A is considered to play an important role in protecting mice from *M. tuberculosis* lung infection.

### Reduction of granulomatous lesion size in the lungs of IL‐17A KO mice after *M. tuberculosis* infection

**Figure 2:**
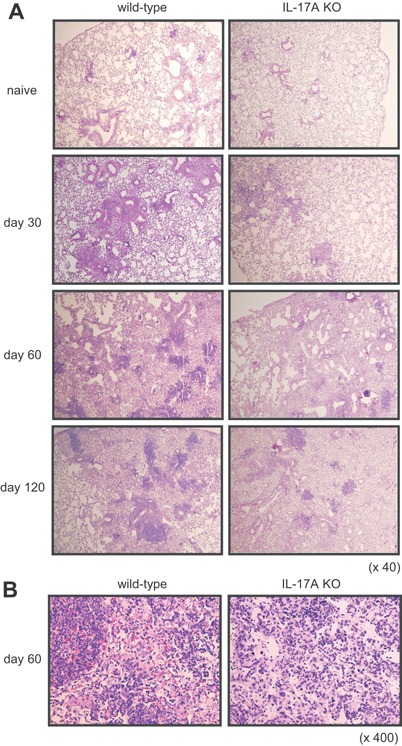
Reduction of the lesion size in the lungs of IL‐17A KO mice after *M. tuberculosis* infection. Wild‐type C57BL/6 or IL‐17A KO mice were inoculated i.t. with *M. tuberculosis* H37Rv. The mice were sacrificed 30, 60, and 120 days after infection, and formalin‐fixed sections were stained with hematoxylin and eosin. Representative lung tissue specimens from the wild‐type C57BL/6 mice (left panel) and the IL‐17A KO mice (right panel) are shown. Magnification, ×40 (A), ×400 (B).

We previously reported that mature granuloma formation is impaired in the lung of the IL‐17A KO mice infected with *M. bovis* BCG 9, 10. However, it was not clear whether the defect impaired the pulmonary granuloma formation at the chronic stage of *M. tuberculosis* infection. To address this issue, we examined the histological changes in the lungs after i.t. infection with *M. tuberculosis* (Fig. 2). Although mycobacterial infection induced histopathological changes in the lungs of both the wild‐type C57BL/6 and IL‐17A KO mice, the granuloma number and size were smaller in IL‐17A KO mice than in wild‐type C57BL/6 mice (Fig. 2A). Furthermore, the granulomas in the lungs of IL‐17A KO mice were less densely packed with mononuclear cells compared to those of wild‐type C57BL/6 mice (Fig. 2B). These data indicate that IL‐17A is important in granuloma formation during *M. tuberculosis* infection.

### Susceptibility to mycobacterial infection is higher in IL‐17A KO mice

**Figure 3:**
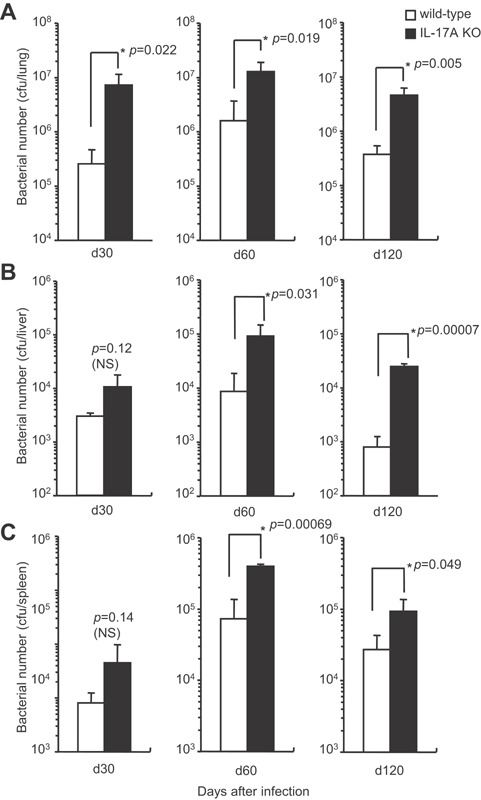
The bacterial growth in various organs of IL‐17A KO mice after mycobacterial infection. Wild‐type C57BL/6 or IL‐17A KO mice were inoculated i.t. with 1 × 10^3^ CFU of *M. tuberculosis* H37Rv, and the CFU in the lungs (A), livers (B), and spleens (C) was determined on days 30, 60, and 120 after the infection. The statistical analysis was performed with Student's *t*‐test. The asterisk (*) indicates that there was a significant difference compared with wild‐type C57BL/6 mice.

To investigate whether the protective immunity against mycobacteria is affected in IL‐17A KO mice, the kinetics of bacterial burden in the lung, spleen, and liver were monitored for 120 days after low dose i.t. infection of *M. tuberculosis* (Fig. 3). The number of bacteria in the lungs of IL‐17A KO mice was significantly higher than that in the wild‐type C57BL/6 mice on days 30, 60, and 120 (*P* = 0.022, *P* = 0.019, and *P* = 0.005, respectively) of the *M. tuberculosis* infection (Fig. 3A). The bacterial number in the liver and spleen of IL‐17A KO mice was also significantly higher on days 60 and 120 after the infection than those in the wild‐type C57BL/6 mice (Fig. 3B and C). These data suggest that IL‐17A is indispensable in the protective immune response that suppresses mycobacterial expansion in the infected organs.

### Identification of IL‐17A‐expressing cells in response to *M. tuberculosis* infection

**Figure 4:**
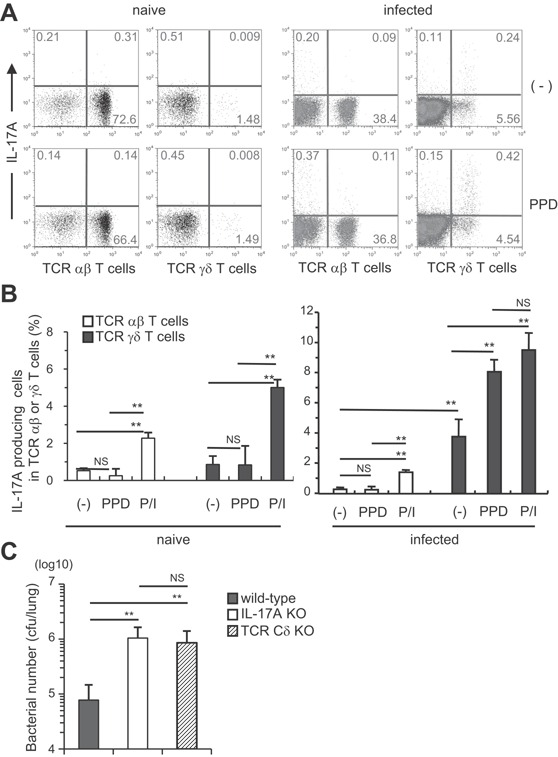
TCR γδ^+^ T cells were the major IL‐17A‐producing cells in the lungs of *M. tuberculosis*‐infected mice. Wild‐type C57BL/6 mice were inoculated i.t. with *M. tuberculosis* H37Rv or left untreated (A and B). The PIF cells (5 × 10^5^ cells) were prepared on day 60, and were cultured with PPD (5 μg/ml) in the presence of naive spleen antigen‐presenting cells (1 × 10^5^ cells) for 18 h at 37°C, and with GolgiPlug for the last 6 h. The cells were also stimulated with PMA and ionomycin. After the culture, the cells were surface stained with FITC‐CD3e, PerCP‐Cy5.5, or APC‐conjugated anti‐TCR Cβ and APC‐conjugated TCR Cδ mAbs. Surface‐stained cells were subjected to intercellular cytokine staining with a PE‐conjugated anti‐IL‐17A mAb. The samples were analyzed by FCM (A, naïve; B, infected). Wild‐type C57BL/6, IL‐17A KO, and TCR Cδ KO mice were inoculated i.t. with 1 × 10^3^ CFU of *M. tuberculosis* H37Rv (C), and the CFU in the lungs was determined on day 60 after the infection. The statistical analysis was performed with ANOVA. Asterisks (*) indicate significant difference between two groups.

We previously reported that TCR γδ^+^ T cells are the major IL‐17A‐producing cells in the *M. bovis* BCG‐infected lungs until day 28 post‐infection 9, 10. To determine whether the IL‐17A‐producing cells were involved in a later stage of *M. tuberculosis* infection, we first analyzed the presence of mycobacterial antigen‐specific IL‐17A‐producing cells in the pulmonary infiltrated (PIF) cells of *M. tuberculosis*‐infected mice on day 60 (Fig. 4A and B; right panels). Unexpectedly, TCR αβ^+^ T cells that produce IL‐17A in response to purified protein derivative (PPD) were hardly detected in the lungs after *M. tuberculosis* infection. Furthermore, IL‐17A production was not detected from the phorbol 12‐myristate 13‐acetate (PMA) plus calcium ionophore ionomycin‐stimulated TCR αβ^+^ T cells, which suggests that Th17 cells are not induced in the *M. tuberculosis*‐infected lungs. On the other hand, the ratio of IL‐17A producing TCR γδ^+^ T cells was significantly higher than that of TCR αβ^+^ T cells after stimulation. A high level of IL‐17A production was detected from the unstimulated TCR γδ^+^ T cells. In addition, we performed FCM analysis of resident lymphocytes in the lung of uninfected wild‐type C57BL/6 mice. TCR γδ^+^ T cells were also detected in the naïve lung although the ratio of the TCR γδ^+^ T cells were lower than that in the infected lung (Fig. 4A and B; left panels). From those observations, we reasoned that IL‐17A‐producing TCR γδ^+^ T cells exist as lung resident lymphocytes, and they expand or are further accumulated in the lung after mycobacterial infection.

Furthermore, we analyzed role of IL‐17A producing TCR γδ^+^ T cells in host defense against chronically infected *M. tuberculosis* using TCR Cδ KO mice, which lack TCR γδ^+^ T cells. On day 60 after infection, the bacterial burden in the lungs of the TCR Cδ KO mice was significantly higher than that of the wild‐type mice, just as observed on the IL‐17A KO mice (Fig. 4C). These data suggest that IL‐17A‐producing TCR γδ^+^ T cells are indispensable arm of protective immunity to mycobacterial infection in the lungs.

IL‐17A has been previously reported to be involved in the immune response against *M. bovis* BCG infection at an early stage of the response 9, 10. These data imply that TCR γδ^+^ T cells are induced from a relatively early stage until the chronic stage of the mycobacterial infection, and are maintained in the lungs.

### BCG vaccination does not induce Th17 cells in the lungs after *M. tuberculosis* challenge

**Figure 5:**
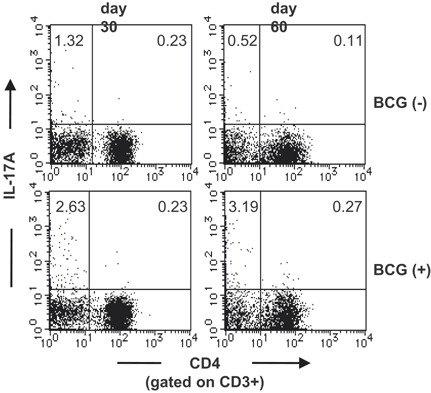
BCG vaccination did not induce Th17 cells in the lungs after *M. tuberculosis* challenge. Wild‐type C57BL/6 mice were vaccinated with *M. bovis* BCG 30 or 60 days before *M. tuberculosis* H37Rv infection. The PIF cells (5 × 10^5^ cells) were prepared on the 4th week after the *M. tuberculosis* infection, and were cultured with PPD (5 μg/ml) in the presence of naive spleen antigen presenting cells (1 × 10^5^ cells) for 18 h at 37°C, and with GolgiPlug for the last 6 h. After the culture, the cells were surface stained with FITC‐CD3 and APC‐conjugated anti‐CD4. Surface‐stained cells were subjected to intercellular cytokine staining with a PE‐conjugated anti‐IL‐17A mAb. The samples were analyzed by FCM.

*M. bovis* BCG is a strong inducer of Th1 immune responses, and vaccination with *M. bovis* BCG suppresses the development of tuberculosis. It has been demonstrated that IL‐17A is required for the generation of Th1 cell responses and host immunity against various pathogens 10, 12, 13. In previous studies, we have shown that IL‐17A was hardly detected from TCR αβ^+^ T cells in the lungs of wild‐type C57BL/6 mice after i.t. *M. bovis* BCG infection at a relatively early stage 9, 10. Th17 cells have protective effects against *M. tuberculosis* infection in vaccinated mice 14, 15. Therefore, we examined whether vaccination with *M. bovis* BCG induces Th17‐cell responses in *M. tuberculosis* infected lungs. CD4^+^ IL‐17A^+^ Th17 cells were not induced by *M. bovis* BCG vaccination before *M. tuberculosis* infection (Fig. 5). On the other hand, a higher ratio of CD3^+^ CD4^−^ T cells present in the lungs of BCG immunized wild‐type C57BL/6 mice produced IL‐17A compared to non‐immunized mice. The IL‐17A^+^ CD3^+^ CD4^−^ cells were TCR γδ^+^ T cells. Therefore, in the case of *M. bovis* BCG vaccination, the IL‐17A‐producing cells were TCR γδ^+^ T cells, not Th17 cells.

### Impaired Th1‐type cytokine production in the lungs of IL‐17A KO mice chronically infected with *M. tuberculosis*

**Figure 6:**
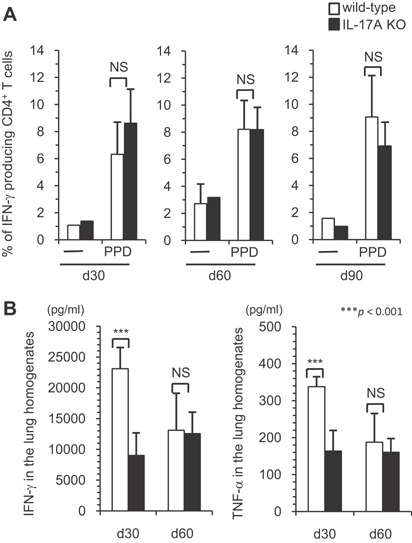
The Th1 immune response in the early phase against *M. tuberculosis* infection. Wild‐type C57BL/6 or IL‐17A KO mice were inoculated i.t. with *M. tuberculosis* H37Rv. Mice were sacrificed 30, 60, and 90 days after infection, and the lung lymphocytes were stimulated with PPD, and the percentages of IFN‐γ producing CD4^+^ cells in the CD3^+^ T cell population were determined using FCM (A). In some experiments, the concentrations of IFN‐γ in the supernatants of the lung lymphocytes were determined by an ELISA using the DuoSet ELISA development kit, according to the manufacturer's protocol (B). The statistical analysis was performed with Student's *t*‐test. Asterisks (*) indicate significant difference between two groups.

Th1‐type cytokines, especially IFN‐γ, are known to play a critical role in the protection against *M. tuberculosis* infection 16, 17, 18. We next investigated whether the absence of IL‐17A affected the antigen‐specific Th1 immune‐response to mycobacterial antigen after i.t. infection with *M. tuberculosis* at chronic stage. On days 30, 60, or 90 after the infection, a similar level of IFN‐γ producing cells in pulmonary lymphocytes was observed in the wild‐type C57BL/6 and IL‐17A KO mice in response to PPD stimulation (Fig. 6A). These data were in agreement with our previous data showing that PPD‐reactive CD3^+^ IFN‐γ^+^ Th1 cells were normally induced in both wild‐type C57BL/6 and IL‐17A KO mice on day 28 of the infection 9, although a decreased total number of CD3^+^ IFN‐γ^+^ T cells was observed in the lungs of the IL‐17A KO mice 10. Therefore, it is possible that the total IFN‐γ production in the *M. tuberculosis*‐infected lungs was decreased in the IL‐17A KO mice. To address this possibility, we examined the production of Th1 type cytokines in *M. tuberculosis*‐infected lungs by ELISA (Fig. 6B). The concentrations of IFN‐γ and TNF in the lung homogenates of IL‐17A KO mice on day 30 after *M. tuberculosis* infection were significantly lower than those of the wild‐type C57BL/6 mice, while the levels were comparable between IL‐17A KO and wild‐type C57BL/6 mice on day 60. These finding imply that IL‐17A participates in the IFN‐γ production at an early phase of *M. tuberculosis* infection.

**Figure 7:**
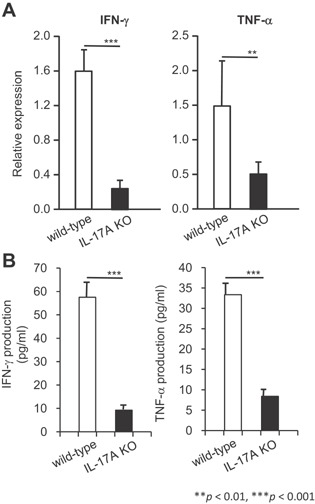
Th1‐type cytokine expression in the lungs of the surviving IL‐17A KO mice after pulmonary *M. tuberculosis* infection. Wild‐type C57BL/6 or IL‐17A KO mice were inoculated i.t. with *M. tuberculosis* H37Rv. The expression levels of IFN‐γ and TNF expression in the infected lungs were analyzed on day 250 after *M. tuberculosis* H37Rv infection by real‐time RT‐PCR (A), and production of IFN‐γ and TNF in the infected lungs was analyzed by the CBA system (B). The statistical analysis was performed with Student's *t*‐test. Asterisks (*) indicate significant difference between two groups.

We next analyzed the Th1/Th2‐type cytokines in the lungs during the chronic phase of *M. tuberculosis* infection by real‐time RT‐PCR and the cytokine bead array (CBA) system (Fig. 7). On day 250 post‐infection, the expression levels of IFN‐γ and TNF in the lungs of IL‐17A KO mice were significantly lower than those of wild‐type C57BL/6 mice (Fig. 7A). The concentrations of these cytokines in the lung homogenates on day 250 were also lower in the IL‐17A KO mice compared to the wild‐type C57BL/6 mice (Fig. 7B). IL‐4 and IL‐10 were undetectable by the CBA system, indicating that the immune response did not shift to a Th2 response. Thus, the influence of IL‐17A on the production of Th1‐type cytokines is stronger in the chronic phase of *M. tuberculosis* infection in the lungs.

## Discussion

In this study, we demonstrate that IL‐17A participates in the maintenance of Th1‐type granulomatous cellular immunity in the *M. tuberculosis*‐infected lungs, and are needed to achieve the control of mycobacterial growth in the chronic phase of *M. tuberculosis* infection.

Our observation on a protective role of IL‐17A is supported by several recent reports. *M. bovis* BCG‐vaccinated mice on day 120 but not on day 21 after the inoculation showed enhanced protective immunity against *M. tuberculosis* challenge and increased IL‐17A‐producing CD4^+^ T cells with co‐expression of TNF and IL‐2 in mice 19, suggesting important role of IL‐17A in *M. bovis* BCG‐induced protective immunity to *M. tuberculosis*. IL‐17‐mediated enhancement of protective immunity against pulmonary mycobacterial infection was also observed in mice with vaccine‐mediated induction of Th17 cells 14. Our data showed involvement of IL‐17A‐producing TCR γδ^+^ T cells in protective immunity against *M. tuberculosis* [10 and this report]. In human, *RORC* mutant patients showed decrease of IL‐17A expression by T cells, which resulted in increased susceptibility to mycobacterial infection with decreased anti‐mycobacterial Th1 response 11. These studies suggest that IL‐17A from both TCRγδ T cells and Th17 cells can enhance Th1‐mediated immune response by modulating the Th1 protective immunity.

It is of interest that IL‐17‐producing TCRγδ T cells is maintained even in the presence of IFN‐γ production by mycobacterial Ag‐specific Th1 cells in the mycobacteria‐infected lung. IFN‐γ plays a central role in the protection against mycobacteria and suppresses IL‐23 production by activated dendritic cells and macrophages, resulting in reduced induction of IL‐17A‐producing CD4^+^ T cells 20. In consistent to the report, we found that *M. bovis* BCG‐infected wild‐type C57BL/6 mice showed very weak Th17 immune responses, while antigen‐specific Th1 immune responses were strongly induced 10. However, IL‐17A‐producing cells were clearly detected in the mycobacteria‐infected lungs and participate in protective immunity, which suggest that the IL‐17A‐production of the TCRγδ T cells is regulated independent of Th1 and support protective immunity.

Although IL‐17A is important in protective immune response to a *M. tuberculosis* infection, the IL‐17A level has to be carefully controlled to prevent pathogenic consequences. It was recently shown that repetitive subcutaneous BCG vaccination in *M. tuberculosis*‐infected mice resulted in an enhanced IL‐17A production by Th17 cells, which is highly pathogenic to cause extensive tissue damage without enhancement of protective immunity 21. It is possible that TCR γδ^+^ T cells can produce optimal level of IL‐17A, which does not damage infected lungs but support Th1‐mediated protective immunity.

Mechanism of IL‐17A production by the TCR γδ^+^ T cells in the mycobacteria‐infected lung of mice is not yet clarified but it is possible signal transduction mediated by TCR or cytokine receptors are involved. We demonstrated that the IL‐17A‐producing TCR γδ^+^ T cells in the mycobacteria‐infected lung express Vγ4 or Vγ6 9, 10, and the repertoire is same as those of IL‐17A‐prodicing TCR γδ^+^ T cells induced by another intracellular bacterial pathogen *Listeria monocytogenes* 22, 23. In addition, IL‐17A‐producing TCR Vγ6^+^ γδ^+^ T cells were induced by infection of extracellular bacteria *Escherichia coli* 24. These results suggest that the Vγ4^+^ or Vγ6^+^ TCR γδ^+^ T cells may recognize some molecule that is widely distributed in bacteria and produce IL‐17A. It was recently reported that CD1d‐restricted lipid Ag‐specific TCR γδ^+^ T cells recognize cardiolipin, which is present in most prokaryotes 25. Therefore, it is possible that Vγ4^+^ or Vγ6^+^ TCR γδ^+^ T cells recognize some bacterial common Ag such as cardiolipin to produce IL‐17A. Alternatively, it is possible that IL‐17A production of the TCR γδ^+^ T cells is determined by expression of cytokine receptors, and inflammatory cytokines such as IL‐23, IL‐1β, IL‐6, and TGF‐β may activate the cells to produce IL‐17A. Further analyses are required to clarify the involvement of TCR or cytokine receptors in the TCR γδ^+^ T cell‐mediated immune response.

Recently, many reports have suggested a broader and more complex role for IL‐17A in various infections. However, the involvement of IL‐17A in protective immunity was not clearly demonstrated in the *M. tuberculosis*‐infected lungs. In the present study, we investigated the involvement of IL‐17A in the immune response against chronic infection with *M. tuberculosis*. Our results indicate that the IL‐17A produced by TCR γδ^+^ T cells is an important cytokine involved in the development of protective immunity against *M. tuberculosis* infection, even at the acquired immunity level. Understanding the interactions of mycobacteria and infected lung tissue may lead to new prophylactic and treatment modalities that have not been considered previously.

## Materials and Methods

### Animals

C57BL/6 mice were purchased from Japan SLC (Shizuoka, Japan). IL‐17A KO mice were generated as previously described 26. TCR Cδ KO mice on a C57BL/6 background were generously provided by Y. Yoshikai (Kyushu University). These mice were bred under specific pathogen‐free conditions in our institute. Eight‐ to 12‐week‐old male mice were used for the experiments. This study was approved by the Committees on Ethics in Animal Experiments and on Safety of Living Modified Organism Experiments in the University of the Ryukyus. Experiments were carried out under the control of the Guideline for Animal Experiments of the University of the Ryukyus.

### Microorganisms and bacterial infection

*Mycobacterium tuberculosis* H37Rv was cultured in a incubator for 3–4 weeks at 37°C in Middlebrook 7H9 medium (BD, Franklin Lakes, NJ) supplemented with albumin‐dextrose‐catalase enrichment (BD). Small aliquots of *M. tuberculosis* suspended in Middlebrook 7H9 medium containing 10% glycerol were stored at −80°C until use. The viable bacterial numbers were determined by plating serially diluted samples on Middlebrook 7H10 (BD) plates supplemented with oleic acid‐albumin‐dextrose‐catalase enrichment (BD). The colony forming unit (CFU) of bacteria was quantified by colony counting on the plates. The bacteria were re‐suspended in PBS before use.

The mice were inoculated intratracheally with 1 × 10^3^ or 1 × 10^5^ CFU of *M. tuberculosis* H37Rv in 50 μl of PBS.

### Bacterial counts in organs

The mice were sacrificed and the lungs, livers, and spleen were removed. The organs were homogenized in saline containing 0.05% Tween 80. Ten‐fold serial dilutions of the homogenates were placed onto Middlebrook 7H10 agar (BD). The plates were incubated at 37°C for 3–4 weeks. After incubation, colonies were counted and the bacterial counts in organs were calculated as the CFU.

### Histopathology

The lungs were fixed in buffered formalin and embedded in paraffin for histopathological examination. Thin sections with 3.5 μm thickness were prepared and stained with hematoxylin and eosin.

### Cell preparation, cell culture, and titration of cytokines

The lung was perfused with PBS through the right ventricle before excision from the mice. The excised lung tissue, separated from all the associated LNs, was minced by a gentleMACS™ dissociator (Miltenyi Biotec, Bergisch Gladbach, Germany) and incubated for 1 h at 37°C in 15 ml of PBS containing 1.0% FBS, 125 U/ml of collagenase I (Sigma, St. Louis, MO), 60 U/ml of DNase I (Sigma), and 60 U/ml of hyaluronidase (Sigma). Single cell suspensions (PIF cells) were prepared by passing through 30‐μm stainless steel mesh. To enrich the pulmonary lymphocytes, PIF cells were re‐suspended in 8 ml of 45% Percoll solution (Amersham Biosciences, Piscataway, NJ), overlayed on 5 ml of 67.5% Percoll solution, and centrifuged at 2200 rpm (800 g) for 20 min at 20°C. The cells at the interface were collected and single‐cell suspensions were prepared in complete RPIM 1640 medium supplemented with 10% FBS. The suspensions (5 × 10^5^ cells in volume of 200 μl complete RPIM 1640 medium) were seeded to each well in 96‐well plates and incubated in triplicate with or without 5 μg/ml of purified protein derivative of *M. tuberculosis* (PPD). The cytokine level in the culture supernatant or supernatants of lung homogenate was determined using a DuoSet ELISA development kit (R&D systems, Minneapolis, MN) for IFN‐γ or TNF. In some experiments, cytokine concentration was determined by cytokine bead array (CBA) Th1/Th2 cytokine kits (BD).

### Quantitative RT‐PCR

Total RNA was extracted from organs using Trizol reagent (Life Technologies, Grand Island, NY). The cDNA was reverse‐transcribed from total RNA with random hexamers and Superscript II reverse transcriptase (Life Technologies). The synthesized first‐strand cDNA were amplified using a StepOnePlus™ real‐time PCR system (Life Technologies). The amplified PCR products were quantified by detecting SYBR Green incorporation. Thermal cycling was initiated with a first denaturation step of 20 sec at 95 °C, followed by 40 cycles of 95 °C for 3 sec and 60 °C for 30 sec. The fluorescence emitted from amplified DNA was read at 60 °C at the end of each cycle. The data of the real‐time PCR amplification were analyzed using the StepOne™ real‐time PCR system software (Life Technologies). The cycle number at which the various transcripts was detectable, referred to as the threshold cycle (Ct), was compared with that of β‐actin and referred to as ΔCt. The relative gene level was expressed as 2^−(ΔΔCt)^, in which ΔΔCt equals ΔCt of the experimental sample minus ΔCt of the control sample. The sequence of primers used are as follows: IFN‐γ sense (5′‐TCA AGT GGC ATA GAT GTG GAA GAA‐3′), IFN‐γ antisense (5′‐TGG CTC TGC AGG ATT TTC ATG‐3′); TNF sense (5′‐CGT GGA ACT GGC AGA AGA G‐3′), TNF antisense (5′‐GTA GAC AGA AGA GCG TGG TG‐3′); β‐actin sense (5′‐TGG AAT CCT GTG GCA TCC ATG AAA C‐3′), and β‐actin antisense (5′‐TAA AAC GCA GCT CAG TAA CAG TCC G‐3′).

### Flow cytometric analysis of intracellular cytokine

To analyze the IL‐17A expression of the cells of the in vivo infection system, PIF cells or pulmonary lymphocytes from *M. tuberculosis*‐infected mice were incubated with or without 1 μg/ml calcium ionophore A‐23187 (Calbiochem, San Diego, CA) and 25 ng/ml phorbol 12‐myristate 13‐acetate (PMA, Sigma) for 6 h at 37°C and 5% CO_2_ in the presence of GolgiPlug (BD). The cells were pretreated with culture supernatant from 2.4G2 hybridoma producing mAb specific for Fcγ receptors II/III (Fc‐blocker), and were then surface‐stained with allophycocyanin (APC)‐conjugated anti‐TCR γδ (eBioscience, San Diego, CA), CD4 (BD), FITC‐conjugated anti‐CD3ϵ (BD), and TriColor‐conjugated anti‐TCR αβ (Caltag Laboratories, Burlingame, CA) mAbs. Surface‐stained cells were subjected to intercellular IL‐17A staining. For intracellular cytokine staining, we used PE‐conjugated anti‐IL‐17A (BD) mAb after permeabilization of the cells using cytofix/cytoperm kits (BD).

To examine the antigen‐specific Th1 immune‐response in the in vivo infection system, pulmonary lymphocytes were incubated with or without 5 μg/ml PPD in the presence of mitomycin C‐treated spleen cells (1 × 10^5^ cells) from naive mice for 24 h at 37°C and 5% CO_2_, with the addition of GolgiPlug for the last 6 h. Cells were pretreated with Fc‐blocker, and subsequently surface‐stained with APC‐conjugated anti‐CD4 and FITC‐conjugated anti‐CD3ϵ mAbs. To detect Th1 cells, we used PE‐conjugated anti‐IFN‐γ (BD) mAb.

For both intracellular IL‐17A and IFN‐γ staining, cells were detected by a FACSCalibur flow cytometer (BD). The data were analyzed with CellQuest software (BD).

### Flow cytometric analysis of cytokine concentration

IFN‐γ and TNF concentrations in the lung lymphocytes were determined with the CBA Th1/Th2 cytokine kits (BD) according to the manufacturer's instructions. In brief, 50 μl of mixed capture beads were incubated with 50 μl samples for 1 h at 25°C, and then 50 μl of mixed PE detection reagent was added. After incubation for 1 h at 25°C in the dark, the complexes were washed twice and analyzed using a FACSCalibur flow cytometer. Data analysis was conducted using the FCAP Array software (BD).

### Statistical analysis

The statistical significance of the data was determined by the generalized Wilcoxon**’**s test for survival rate. Student's *t*‐test and one‐way ANOVA of the comparisons of bacterial numbers in the lung were used for the other analyses. A *P*‐value of <0.05 was considered to indicate a statistically significant difference.

## Conflict of Interest

None declared.
